# The phasor-FLIM fingerprints reveal shifts from OXPHOS to enhanced glycolysis in Huntington Disease

**DOI:** 10.1038/srep34755

**Published:** 2016-10-07

**Authors:** Sara Sameni, Adeela Syed, J. Lawrence Marsh, Michelle A. Digman

**Affiliations:** 1 Laboratory for Fluorescence Dynamics, UC Irvine, CA, USA.; 2 Department of Biomedical Engineering, UC Irvine, CA, USA.; 3 Department of Developmental and Cell Biology, UC Irvine, CA, USA.; 4 Department of Chemical Engineering and Material Sciences, UC Irvine, CA, USA.

## Abstract

Huntington disease (HD) is an autosomal neurodegenerative disorder caused by the expansion of Polyglutamine (polyQ) in exon 1 of the Huntingtin protein. Glutamine repeats below 36 are considered normal while repeats above 40 lead to HD. Impairment in energy metabolism is a common trend in Huntington pathogenesis; however, this effect is not fully understood. Here, we used the phasor approach and Fluorescence Lifetime Imaging Microscopy (FLIM) to measure changes between free and bound fractions of NADH as a indirect measure of metabolic alteration in living cells. Using Phasor-FLIM, pixel maps of metabolic alteration in HEK293 cell lines and in transgenic Drosophila expressing expanded and unexpanded polyQ HTT exon1 in the eye disc were developed. We found a significant shift towards increased free NADH, indicating an increased glycolytic state for cells and tissues expressing the expanded polyQ compared to unexpanded control. In the nucleus, a further lifetime shift occurs towards higher free NADH suggesting a possible synergism between metabolic dysfunction and transcriptional regulation. Our results indicate that metabolic dysfunction in HD shifts to increased glycolysis leading to oxidative stress and cell death. This powerful label free method can be used to screen native HD tissue samples and for potential drug screening.

The mechanisms underlying neurodegeneration in Huntington Disease(HD) are unknown, but compelling evidence suggests that mitochondrial defects may play a central role^1^. Mitochondria dysfunction has been linked to a range of disease and health problems such as cancer and neurodegenerative diseases^2^. Thus early detection of mitochondria anomalies and metabolic dysfunction might help contribute to effective diagnosis and subsequent treatment.

Previous studies show that HD onset can be identified from disturbed glucose metabolism. Glucose metabolism in asymptomatic HD carriers reveals significant hypometabolism and a 2–3% reduction in caudate glucose metabolism per year. This implies there is a significant reduction of glucose metabolism occurring during the onset of the disease, and the measurement of striatal metabolism receptor binding would be a good marker for the diagnostic of the progression of the disease^3^,^4^. Yet other studies report that lactate produced by glucose uptake is enhanced in HD brain tissue of transgenic and 3-nitropropionic acid-treated mouse models for HD^5^. Olah and coworkers measured high glycolytic flux in the posterior region of HD brains by measuring mitochondrial complex activities, NADH absorption, pyruvate formation and lactate production^5^. In their experiment-based mathematical model, the authors calculate the increased flux through the glycolytic pathway where they revealed that the increased energy metabolism enhances the rate of glycolytic flux in comparison to the control. However, the hypothesis that the ATP production through oxidative phosphorylation (OXPHOS) indicates a shift metabolism to glycolysis in HD is not yet clear^6^.

Chance *et al*., in the 1950s, pioneered the measurement and quantification of tissue and cellular metabolic states, and since then many studies followed^7^,^8^,^9^. These investigations were based on florescence emission of reduced nicotinamide adenine dinucleotide (NADH) as an endogenous florescent biomarker. NADH is the principal electron acceptor in glycolysis and electron donor in oxidative phosphorylation^10^. While NADH fluoresces, its oxidized form (NAD+) does not, and research has been focused on measuring the fraction of intracellular free and bound forms (to enzymes) of NADH^11^. Given that the fluorescence lifetime of NADH shifts in the bound versus free state, NADH autofluorescence has been used as a biomarker for oxidative stress in alpha-synuclein aggregation, metabolic changes in Wnt signaling in colon cancer was characterized using NADH-FLIM^12^,^13^,^14^. Since FLIM is independent of concentration, the lifetimes of free and bound forms of NADH are unbiased from endogenous levels produced in living cells. Also the lifetime of free NADH is shorter (~0.4 ns), indicating more glycolysis, compared to the fully bound form (3.2 ns–3.4 ns), indicating more oxidative phosphorylation. Thus their fractional contributions can be a measure of oxidative stress or cellular homeostasis^2^,^12^,^15^.

In this work, we have used the phasor approach to FLIM to measure fraction of free to bound NADH as a way to map the metabolic alteration in cells transfected with expanded and unexpanded polyQ HTT exon 1. Image segmentation to isolate the cytoplasm was also done and further characterized a lifetime shift towards the free fractional component of NADH in the cell nucleus. In addition we have performed FLIM in the late third instar eye discs of a Drosophila model of Huntington disease^16^. During this late third instar stage (approximately 3–4.5 days post fertilization) eye imaginal discs complete development and photoreceptor neurons are born^17^,^18^.

Our results indicate that, in the presence of the expanded Huntingtin repeat, fingerprints of the phasor distributions shift towards an increased free NADH lifetime in the nucleus. It has been shown that NADH interaction with regulatory proteins such as CtBP and Sirtuins influences their transcriptional activity^19^,^20^. Moreover, while NAD(P)H is shown to enhance heterodimerisation and DNA binding to transcription factors, its oxidized form, NAD(P), inhibits such activities^20^. Thus we hypothesize that such an increase in nuclear NADH leads to transcriptional dysregulation. Further, we observed an increase in the ratio free/bound NADH (shift toward anaerobic glycolysis) in the cytosolic region in the presence of the expanded Huntingtin repeat. This is in correlation with previous studies that showed hypoxic conditions by a shortening of NADH fluorescence lifetime (i.e. more free NADH) in cells^21^, tissue^22^ and organs *in vitro*^23^ and *in vivo*^24^.

## Results

### Increase free NADH in HEK 293 cell lines expressing the expanded polyQ Exon1

Using HEK 293 cells transiently transfected with exon 1 of the human Huntingtin protein with either unexpanded or expanded polyQ, we show that the relative ratio of free to bound NADH increases on average when cells are challenged with expanded polyQ HTT exon1. This increase in the free/bound ratio indicates that cells expressing the expanded HTT repeat show a shift of ATP production from OXPHOS to glycolytic metabolism. We have used the FLIM phasor approach to in which the fluorescence decay at each pixel is transformed into a single point in the phasor plot, defined in the materials and methods section and in Supplemental Figure S1. In the phasor plot, the s(ω) and g(ω) coordinates for every pixel of the image, Fourier sine vs. the Fourier cosine, are plotted on the y and x axis where the x coordinate spans from 0 to 1 and the y spans from 0 to 0.5. Thus the g coordinate is more sensitive to the ratio of free to bound NADH as shown in supplemental (Figure S2)^25^,^26^.

**Figure 1:**
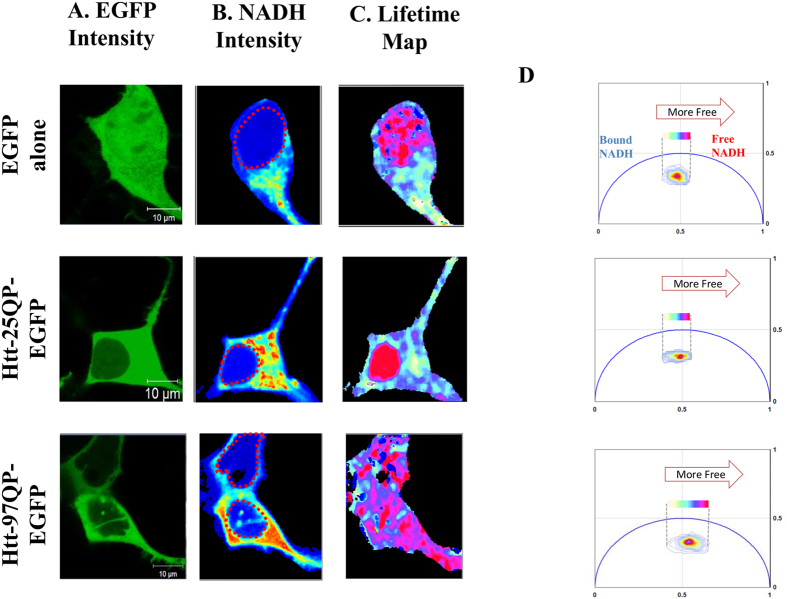
Panel A shows confocal images obtained using the 488 nm laser to excite EGFP directly. In panel B NADH emission was obtained with 740 nm two-photon excitation. NADH is detected with a blue filter (442/46 nm) in one channel and EGFP emission with a green filter (520/35 nm). Nuclei of the cells are shown with dashed red circle. Panel C represents lifetime maps of NADH colored according to the color scale in the phasor plot in panels D. When cells express the expanded Htt 97Qp, there is a significant shift toward the shorter lifetime indicating a higher glycolytic state. Panel D also shows the phasor plot for each representative cell. The phasor histogram of the cell transfected with 97Q is shifted to the direction of free NADH. Scale bar on the image is 10 μm.

**Figure 2:**
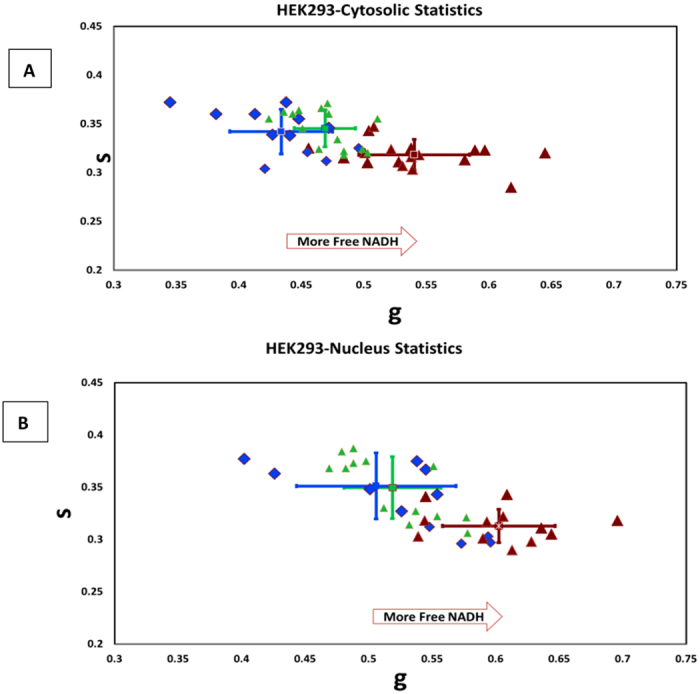
(**A**) Scatter plot of NADH cell phasor FLIM in the cytoplasmic pool of the Httex1p25Q (N = 12, in blue) expressing cells compared to the Httex1p97Q cells in red (N = 18). EGFP control is plotted in green triangle (N = 15). Each point represents the average lifetime plotted in the phasor coordinates. Using image segmentation the cytoplasmic region for each cell is selected. Expanded cells (97Q) shows higher free to bound NADH ratio (shifted toward right). (**B**) Similar plot was obtained for individual cell nuclei of the Httex1p25Q cells compared to Httex1p97Q cells. Cells transfected with 97Q are further shifted towards the right side of the plot indicating an increase of free NADH in the nucleus.

Figure 1 shows the simultaneous intensity of EGFP, excited directly at 488 nm, and NADH autofluorescence, excited via two-photon excitation at 740 nm, for HEK 293 expressing EGFP alone, 25QHTT-GFP and 97QHTT-GFP. The color of the lifetime maps (Fig. 1C) corresponds to the fluorescence lifetime distribution along the phasor plot shown in panel D for each of the 3 conditions. We applied image segmentation to isolate the nuclear compartment from the cytoplasmic pool as is shown in supplement S3 to further quantify the average phasor coordinates corresponding to the g and s axis on the phasor plot. Our results indicate a significant shift in NADH lifetime, indicating a transition from the NADH bound to the NADH free form, in cells expressing the expanded Httex1p97Q-EGFP protein compared to the Httex1p25Q expressing cells as shown in Fig. 2A (P = 1.02 × 10^−6^).

We identified two distinct populations (Fig. 2) in the nuclear and cytoplasmic phasors corresponding to the Httex1p25Q-EGFP and Httex1p97Q-EGFP variants in HEK 293 cells. To further confirm our data and to ensure that changing the fluorescent protein does not perturb NADH readings we have performed experiment in 15 more animals (total of 66 ROIs) tagged with Kaede (Figure S4). The applied image segmentation for the nuclear compartment shows a decreased nuclear activity in cells expressing the expanded form of the HTT protein. In both expanded and unexpanded HTT protein expressing cells, the nucleus is characterized by high amount of free NADH. This is consistent with the results published by Wright *et al*.^12^ which shows reduced nuclear activity characterized by shorter lifetime (more free NADH) for undifferentiated or early differentiating cells and more bound NADH due to increased nuclear activity in differentiating cells. In the expanded HTT expressing cells there is a significant increased fraction of free NADH indicating a lower nuclear activity (p = 0.0024) (Fig. 2B). No differences were observed for the control experiments of the cell cytoplasm and nucleus in the EGFP expressing cells compared to the wild type, Httex1p25Q-EGFP with p value higher than 0.05 (p = 0.206 and p = 0.209 accordingly).

### Transgenic Drosophila results

To further test our results *in vivo*, we have examined the eye discs of a transgenic Drosophila model of HD. Drosophila is an excellent source for modeling neurodegenerative disease as it contains fully functional nervous system with separate specialized functions including vision, olfaction, learning and memory^16^. The most common method for generating restricted transgene expression in Drosophila is based on the yeast GAL4 protein and its target upstream activating upstream sequence (UAS) system^27^.

The eye develops from the eye antennal disc. The disc arises from about 20 cells of the optic primordium in the embryonic blastoderm^28^. By the third instar, the eye disc forms approximately 20,000 cells 28. Barbaro *et al*. earlier showed temporal gradients of neurons expressing HTT transgenes in Drosophila eye discs. Cells begin to differentiate into neurons in the eye disc as a wave of differentiation passes from posterior to anterior during L3 (defined by the morphogenetic furrow). As the wave moves toward the anterior in an array of cells ordered in rows forward, each row of cells from the leading edge of the furrow toward the posterior has been expressing the HTT transgene 2 h longer than the row in front of it^29^. We have selected to analyze the posterior part of the eye disc where the oldest cells expressing the transgenes (120Q, 96Q or 25Q) reside.

**Figure 3:**
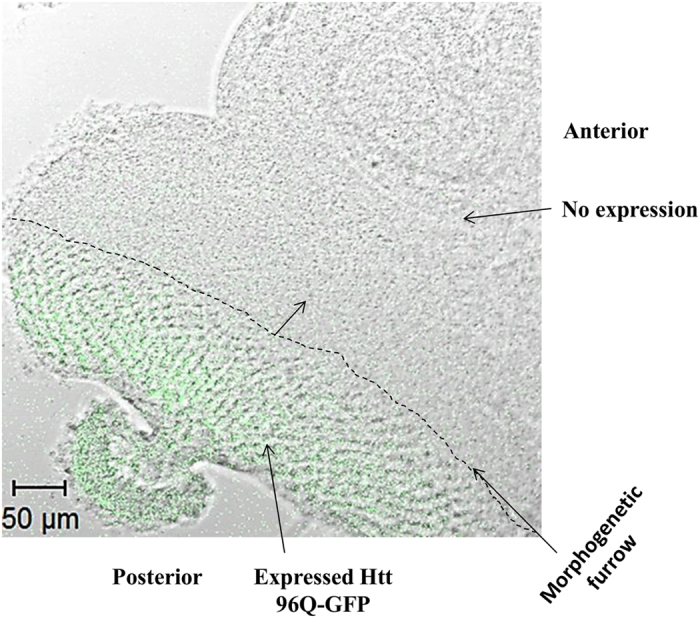
Tiled images of ELAV GAL4 X UAS Httexon1p Q96-GFP Drosophila eye disc. Bright field image merged with FITC shown here (Scale bar on the image is 50 um).

**Figure 4:**
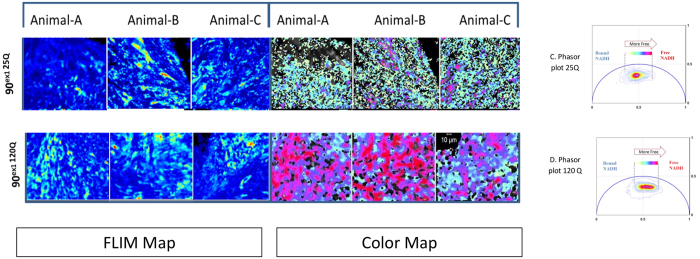
Unexpanded ELAV-GAL4 X UASQ25 vs. expanded ELAV-GAL4 X UASQ120 expressing flies results are depicted here. NADH emission obtained with 740 nm two-photon excitation is shown under the intensity map. Lifetime color map of NADH is depicted on the right (color map). Shorter lifetime is color coded with red here while longer lifetime on the phasor plot is associated with cyan. As it is shown here Htt 120Q cells are characterized with a distinct population shifted toward shorter lifetime (red) that has a higher ratio of free to bound NADH. Panels C&D show the phasor plot for 25Q and 120 Q respectively. The phasor histogram is shifted toward the direction of free NADH (glycolysis) in the expanded form 120Q compared to unexpanded 25Q. Scale bar is 10 um.

Figure 3 shows tiled image of expanded 90^ex1 96Q-GFP^. The Drosophila eye disc excited at 488 nm in respect to the whole eye disc (bright field image merged with FITC) is depicted here. The posterior region expressing the HTTexon1p 96Q-GFP was selected and the distributions of lifetimes were mapped. Similar to what we have observed in the HEK 293 cells, a shift toward shorter lifetime in cells expressing HTT 96Q-GFP of the live eye disc was detected. To ensure that the expression of GFP did not perturb the metabolism in this model, we tested the unexpanded versus the expanded HTT with no fluorophore tag (Unexpanded 90^ex1 25Q^ vs. expanded 90^ex1 120Q^ expressing flies). The images here are obtained from the most posterior part of each eye disc that is associated with either 25Q or 120Q expressions. As shown in Fig. 4, the three representative tissue ROI of the 25Q populations are characterized with longer lifetime associated with decreased ratios of free to bound NADH similar to what was observed in the HEK 293 cell line (white/aqua color) with unexpanded transfection; while the expanded models (i.e. 120 Q) are shifted towards the shorter lifetime with increased ratio of free to bound NADH (pink/red color).

**Figure 5:**
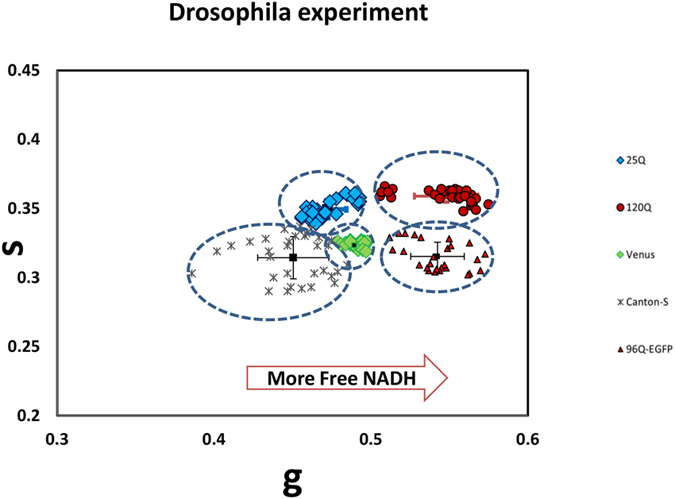
Scatter plot of the NADH phasor FLIM showing average g and s phasor values for each animals eye disc ROI (N) for total of 15 animals and a total of N = 149 ROI measurements. The blue diamond refers to 25Q (N = 30), green diamond Venus control (N = 28), gray asterisk wildtype with no HTT (Canton-S, N = 32).Tissue with expanded expression 120Q (N = 32, in red circle) and 96Q-EGFP (N = 27, in red triangles) indicates shortening of the lifetime towards the glycolytic state, shifted to the right.

Figure 5 shows the scatter plot of phasor lifetimes averages for the eye discs expressing the unlabeled unexpanded (blue) and expanded HTT protein (red). The analysis of the phasor distribution in this case is performed by cluster identification as described in ref. 30. The clusters refer to all the pixels selected in the ROI and are the average coordinates lifetime for each ROI of the three eye discs.

The Student t test was done to compare the 25Q population and 120Q population in terms of g values. Our result indicates that these two populations are statistically significantly different (p-value of 0.034). In addition, the 25Q group shows significantly longer lifetime compared to 96Q group (p-value 0.023).

**Figure 6:**
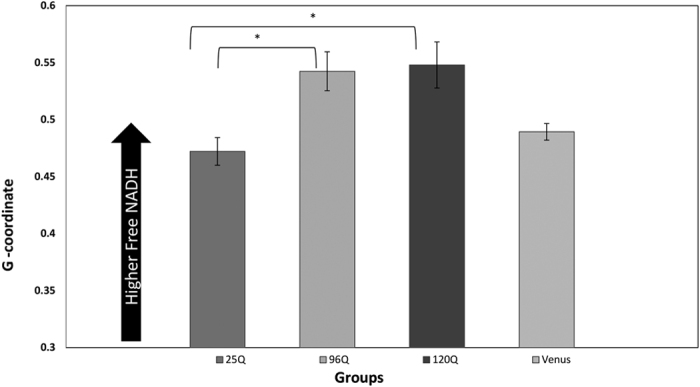
Summary of the data analysis for live tissue of drosophila eye disc (expanded 96Q (N = 27), 120Q (N = 32), unexpanded 25Q (N = 30), and control (Venus, N = 28) are depicted here. Arrow bar shows the direction of Free NADH. Expanded Htt(both 96Q and 120Q) are characterized with significant increased ratio of free to bound NADH(more Glycolytic) compared to unexpanded Htt (25Q).

No difference was observed in control group (Venus with no HTT) compared to 25Q with the p-value >0.05(P = 0.14) (Fig. 6).

## Discussion

Energy production deficiency contributes to the progression of HD and a common side effect is weight loss in HD patients^31^. The dysfunction in energy metabolism, in particular mitochondrial perturbation, is a common trend in Huntington pathogenesis studies in human and animal models^32^. It has been postulated that HTT perturbs the activation of SIRT 3 leading to a decrease in concentration of NAD and PGC-1α (a transcriptional coactivator controlling mitochondrial biogenesis and function)^33^. Given that SIRT 3 acetylates the flavoprotein (Fp) subunit of complex II, the loss of SIRT 3 activity in HD leads to a reduced binding of complex II with the Fp thereby dysregulating mitochondrial function^33^.

Alterations of metabolism have recently gained traction in identifying the mechanism by which the metabolic pathway is affected. Insight into metabolic requirements in distinct populations of striatal versus cortical neuron is provided by Gorarne and co-workers^34^. They showed that early subtle defects in glycolysis were found in striatal neurons using PET in living patients. However, contradicting results were recently reported by Nambron *et al*. that revealed metabolic carbohydrate, lipid and protein metabolites as wells as hormones related to energy metabolism screening in HD patients resulted in no significant changes compared to the control subjects^35^. These conflicting results can be due to sensitivity of sample preparation as well as average population or bulk measurements. Thus, pursuing the quest to understand and learn about metabolic dysfunctions in a non-invasive manner and at the single cell level may elucidate the role of impaired metabolic pathways that can lead to transcriptional dysregulation in the disease.

Previous studies in animal models and HD patients reported perturbation of metabolism in HD in animal studies and HD patients. Several studies associate HD with Diabetes Mellitus as significant numbers of R6/2 mice develop diabetes gradually^4^,^36^. Other studies also reported that glucose metabolism can be an indicator of HD onset^37^,^38^.

Our present study, using 2P-FLIM, indicates a decrease in fluoresce lifetime of NADH that is correlated with an increased in free to bound ratio of NADH in HD. These changes indicate ATP production shifts from oxidative phosphorylation to glycolysis that can lead to an increase in oxidative stress and eventual cell death. Given that the lifetimes of NADH and Nicotinamide Adenine Dinucleotide Phosphate (NAD(P)H) are virtually indistinguishable, alternation of free to bound NAD(P)H could also reflect these changes. Our results are consistent with an earlier report by Valencia *et al*. showing elevated NADPH oxidase activity leading to over production of ROS and cell death in HD mice^39^.

Here we were able to examine changes in the fluorescence lifetime of NADH indicating an increased glycolytic state in HD using Phasor FLIM approach. Earlier, Plotegher *et al*. showed shifts of NADH to shorter lifetime (toward the glycolytic state) in the presence of alpha-synuclein aggregates in Parkinson disease (in HEK293-aS)^14^. Similar results were also reported recently using 2P-FLIM by Sandeep *et al*. in a pc12 model of Parkinson disease^40^. More recently in a pilot study, Jentsch used FLIM based technology in opthalmology based setting to examine the alteration in retina of Alzheimer disease patients. In this study, dependence of the fluorescence lifetime imaging parameter on the severity of Alzheimer’s disease is reported^41^.

The real advantage of the powerful method that we have proposed here is that it can be applied in tissue and a pixel map of cellular metabolic alteration can be obtained. We foresee that such precise measurements using phasor-FLIM finger printing approach can help address some of the current issues in the literature.

## Conclusions

The underlying mechanism that affects neurodegeneration in Huntington disease is still under investigation. Compelling evidence indicates that mitochondria may play an essential role that affects neurodegeneration in Huntington disease. In this work, we have measured metabolic perturbations in cells transfected with various glutamine repeats (expanded vs unexpanded) as well as in the transgenic Drosophila eye discs using FLIM coupled with phasor analysis. We used the autofluorescence lifetime of NADH as an intrinsic biomarker for such measurements. Results obtained here indicate a shift toward a larger free/bound NADH ratio in the presence of the expanded Huntington repeat both *in vitro* in Human Embryonic Kidney (HEK 293) cells as well as in the Drosophila eye disc model of Huntington disease. Such a shift in the expanded model may indicate depletion of ATP production and an increase in oxidative stress that can eventually lead to cell death. In addition, nuclear FLIM analysis on expanded polyQ HTT exon1 expressing cells indicates increased free NADH which influences CtBP and Sirtuin activity in the nucleus^43^. We propose that such a shift toward increased free NADH in the nucleus indicates transcriptional dysregulation which is crucial in Huntington pathogenies.

This report fingerprints metabolic shifts from OXPHOS to glycolysis in HD cells. In addition, we show that there is an increase in free NADH in the nuclear compartment in HEK293 expressing the expanded form of the polyQ HTT exon1. This result may indicate a transcriptional dysregulation in the nucleus which is important to understand in HD studies and also for similar neurodegenerative disease such as Alzheimer’s, Parkinson, ALS, and etc. Further investigations need be done to understand if different stages of inclusion formation or inclusions themselves uniquely alter redox state of the cells.

## Material and Methods

### Cell cultures and transfection

Human Embryonic Kidney cell 293(HEK293) plated on 3 ug/ml fibronectin were plated with ~70% confluency and transfected overnight with Httex1p containing pcDNA with varying length of polyglutamine fused at C-terminus to EGFP(Httex1p 97Q-EGFP, Httex1p25Q -EGFP, and EGFP alone).

### Confocal Imaging

The EGFP tagged cells were visualized via confocal microscopy Zeiss LSM 710 confocal microscope (Carl Zeiss, Jena, Germany). Images are obtained with a 488 nm argon-ion laser and imaged using the internal detectors using a filter 500–550 nm. Using laser scanning confocal microscope, the fluorophore of the entire specimen were acquired and assigned a pseudo-color. Images are acquired using 63x oil objective.

### FLIM set up

Fluorescence lifetime imaging were acquired using the same Zeiss LSM 710 confocal microscope equipped with Ti-Sapphire laser (Mai Tai Spectra-Physics, Newport, CA), using external detectors (H7422P-40, Hamamatsu Corporation, Bridgewater, New Jersey) and an ISS A320 FastFLIM unit (ISS, Champaign, IL). NADH fluorescence lifetime was measured upon 2–photon excitation at 740 nm. A 495 nm long-pass filter was used to separates the blue and the green fluorescence channel. NADH emission was detected with a blue filter (442/46 nm) in one channel and EGFP emission with a green filter (520/35 nm). Images were acquired using 63X oil objective. Images were collected with 256 × 256 pixels with a minimum of 100 counts per pixel which requires at least integrating 30 frames with a pixel dwell time of 25.6 us/pixel. The temperature was set at 37 °C throughout the experiment with 5% CO_2_.

### Data analysis

The phasor analysis was performed using the method explained by Digman *et al*.^42^ and analyzed with the SimFCS software available at (www.lfd.uci.edu). Briefly, for each pixel in an image, a phasor lifetime is plotted in a graph using Fourier transformation, we then use a cursor to highlight the cluster of points that corresponded to the lifetime in that range. Figure S1 in supplemental material schematically shows the transformation process to a single point in a 2D phasor plot. Phasor plot coordinates are g and s (x and y).

### Statistical analysis

We have used student’s t-test with critical p-value set at 0.05 for our analysis. Total of 45 HEK293 cells have been tested for *in vitro* study of HD. For the animal study we have used total of 15 animals and analyzed N = 149 total regions of interests (ROI~30–50 μm^2^ in the posterior area of the eye disc)) (N = 30 for 25Q, N = 32 for 120Q, N = 27 for 96Q-EGFP, N = 28 for Venus, N = 32 for wildtype Canton-S). A total of 15 animals which expressed HTT 25Q versus 120Q tagged to a fluorescent protein, Kaede. shows that tagging the protein does not perturb the NADH fluorescence lifetime changes observed in the unlabeled animals (figure S4). For the 120Q-Kaede we have analyzed N = 39 regions of interest of 8 animals imaged at the distal end of the eye disc in comparison with the 25Q-Kaede where N = 27 ROI of 7 animals were analyzed.

### Animal Study

ELAV-GAL4 is used for expression of UAS transgene. Fly stocks were cultured on cornmeal medium at 25 degree (room temperature). Larvae at the late 3^rd^ instar, instar refers to larvae stage, were selected and transferred into a petri dish with dissection buffer. Using forceps, the mouth hooks were pulled away from the rest of the body. The eye discs were dissected away from the rest of the tissue and immediately placed on a coverslip for imaging. In this experiment we have used Unexpanded 90^ex1 25Q^, expanded 90^ex1 96Q-EGFP^ (a kind gift of T. Littleton), expanded 90^ex1 120Q^, and wild type, Canton-S, with no HTT transfection and Venus as controls. In addition we have verified our analysis by co-expressing Kaede expanded vs. unexpanded HTT(90^ex1 25Q-Kaede^ vs. 90^ex1 120Q-Kaede^) to further verify the NADH FLIM readings.

## Additional Information

**How to cite this article**: Sameni, S. *et al*. The phasor-FLIM fingerprints reveal shifts from OXPHOS to enhanced glycolysis in Huntington Disease. *Sci. Rep*. **6**, 34755; doi: 10.1038/srep34755 (2016).

## Supplementary Material

Supplementary Information
