# Supplementary material for: The phasor-FLIM fingerprints reveal shifts from OXPHOS to enhanced glycolysis in Huntington Disease
Source: Sci Rep. 2016 Oct 7;6:34755. doi: 10.1038/srep34755 (PMC5054433; doi:10.1038/srep34755)
Supplement: Supplementary Information [file srep34755-s1.pdf]

Supplements

The phasor-FLIM fingerprints reveal shifts from OXPHOS to enhanced glycolysis in Huntington Disease

Sara Sameni[1], Adeela Syed[2], J. Lawrence Marsh[2], and Michelle A. Digman[1,2,3]

[1]Laboratory for Fluorescence Dynamics, Department of Biomedical Engineering, [2]Department of Developmental and Cell Biology, [3]Department of Chemical Engineering and Material Sciences, University of California Irvine

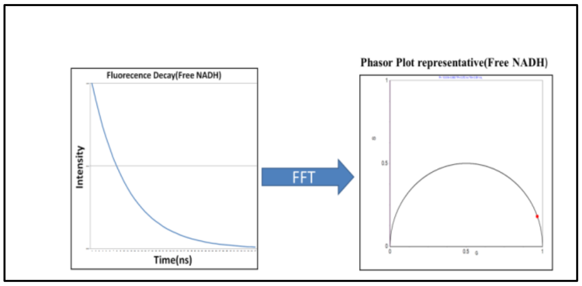

$$g_{i,j}(\omega) = \frac{\int_0^\infty I_{i,j}(t)\cos(\omega t)dt}{\int_0^\infty I_{i,j}(t)dt},$$
$$s_{i,j}(\omega) = \frac{\int_0^\infty I_{i,j}(t)\sin(\omega t)dt}{\int_0^\infty I_{i,j}(t)dt},$$

Figure S1: The process of Phasor transformation is depicted here. Coordinates g and s were obtained using the formulas in the right panel. ω is the angular modulation frequency obtained as ω=2πf where f is 80 MHz for our laser system.

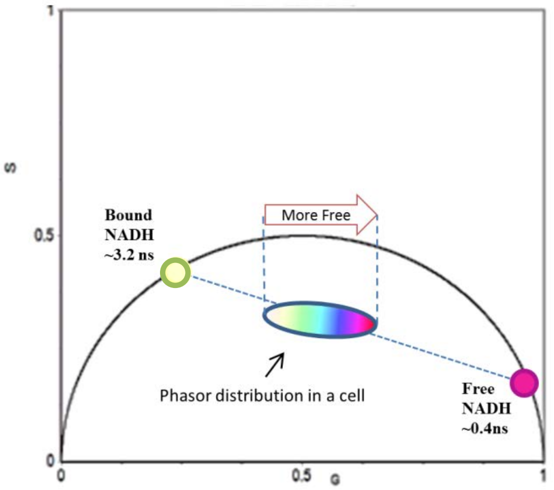

Figure S2: Phasor plot is obtained by calculating the cosine and sine transform of the fluorescence decay at each pixel of an image (Digman et al, 2008). And thus the measured florescent decay is transformed to single points in the 2D phasor with g and s coordinates(x and y axis corresponding to cosine and sine components. The pure NADH is shown in pink circle with a lifetime of 0.4ns The NADH phasors shifts towards the longer lifetime (3.2ns) when free NADH is incubated with lactate dehydrogenase (LDH) depicted by the green/white circle. The dotted blue line connecting Free (pink circle) to the bound NADH (green/white circle) is the total possible range. Due to the linearity rule of phasor coordinate, the cluster of data shown as a mixture of free and bound NADH lays on the straight (blue line) line connecting the pure free to bound NADH.

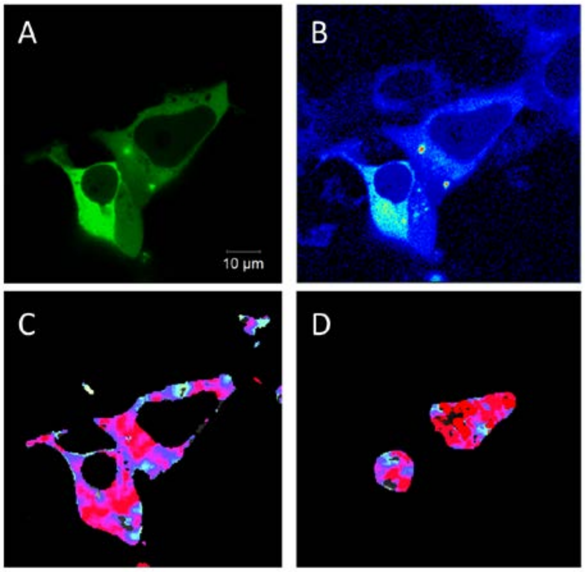

Figure S3: A shows confocal images of 97Q-EGFP using 488 nm. B is the intensity FLIM map obtained with two-photon excitation at 740 nm. C&D shows the segmented image that isolate cytoplasmic portion(C) from nuclear compartment (D).

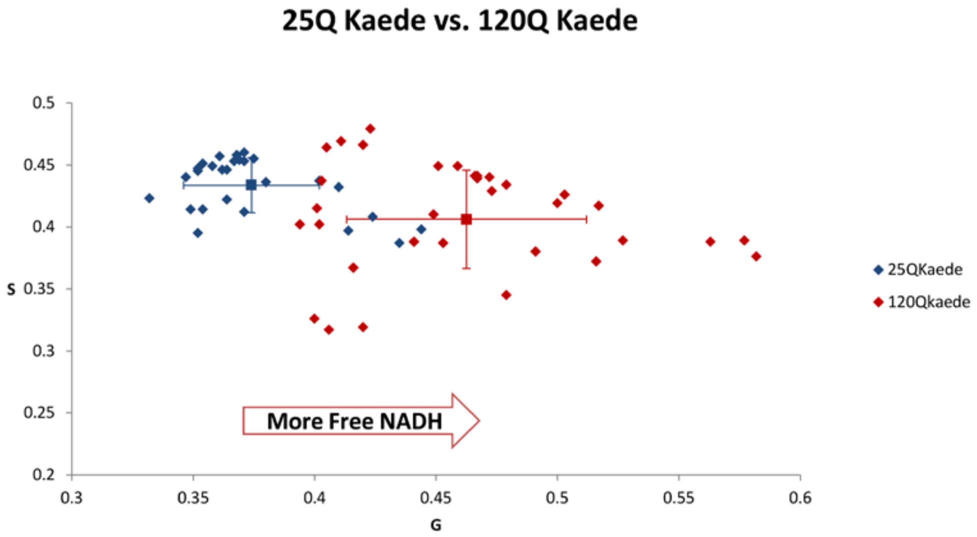

Figure S4: NADH phasor FLIM scatter plot of 25QKaede vs. 120Q kaede showing average g and s phasor values for each animal eye disc ROI for total of 15 animals and 66 ROI measurements. The blue diamond refers to 25Q Kaede(N=27), and expanded expression 120QKaede (N=39, in red diamond) that indicates shortening of the lifetime towards the glycolytic state, shifted to the right similar to the other drosophila experiments that we reported here.
